# The relationship between keratin 18 and epithelial-derived tumors: as a diagnostic marker, prognostic marker, and its role in tumorigenesis

**DOI:** 10.3389/fonc.2024.1445978

**Published:** 2024-10-22

**Authors:** Jiazhi Yan, Aiwei Yang, Shuo Tu

**Affiliations:** ^1^ Queen Mary School, Jiangxi Medical College, Nanchang University, Nanchang, Jiangxi, China; ^2^ School of Basic Medical Sciences, Jiangxi Medical College, Nanchang University, Nanchang, Jiangxi, China

**Keywords:** keratin 18, epithelial-derived tumors, intermediate filament, diagnostic marker, prognostic marker, tumorigenesis

## Abstract

As a structural protein, keratin is mainly expressed in epithelial cells and skin appendages to provide mechanical support and external resistance. The keratin family has a total of 54 members, which are divided into type I and type II. Two types of keratins connect to each other to form keratin intermediate filaments and participate in the construction of the cytoskeleton. K18 is a non-hair keratin, which is widely expressed in simple epithelial tissues with its partner, K8. Compared with mechanical support, K8/K18 pairs play more important roles in biological regulation, such as mediating anti-apoptosis, regulating cell cycle progression, and transmitting signals. Mutations in K18 can cause a variety of non-neoplastic diseases of the visceral epithelium. In addition, the expression levels of K18 are frequently altered in various epithelial-derived tumors, especially adenocarcinomas, which suggests that K18 may be involved in tumorigenesis. Due to the specific expression pattern of K18 in tumor tissues and its serum level reflecting tumor cell death, apply K18 to diagnose tumors and predict its prognosis have the potential to be simple and effective alternative methods. However, these potential roles of K18 in tumors have not been fully summarized. In this review, we focus on the relationship between K18 and epithelial-derived tumors, discuss the value of K18 as a diagnostic and prognostic marker, and summarize the interactions of K18 with various related proteins in tumorigenesis, with examples of simple epithelial tumors such as lung, breast, liver, and gastrointestinal cancers.

## 1 Introduction

Just as the beams are built to maintain the stability of the house in architecture, the cytoskeleton as the “beam of the cell” plays an extremely important role in maintaining the basic morphology of eukaryotic cells. In contrast to fixed beams, the cytoskeleton is a continuously dynamic structure which changes shape by disassembly and reassembly to adapt different requirements. Based on physicochemical properties like diameter, stiffness and dynamics, the polymers that make up the cytoskeleton are mainly divided into three types: microfilaments, microtubules, and intermediate filaments (1). The cytoskeletal network formed by the interaction of the three polymers is not only responsible for maintaining cell stiffness and resisting external forces, but also participates in many important cell life activities, such as the separation of chromosomes during mitosis, the transport of intracellular substances, the perception of external environment, the contraction and relaxation of muscle cells, and the extension of axons and dendrites of nerve cells (2–5). Due to the vital roles of cytoskeleton in cell structure and life activities, the perturbations of three polymers are usually associated with the occurrence and development of various diseases, such as the proliferation and metastasis of tumor cells, the formation of inclusion body in Alzheimer’s disease, and some genetic diseases (6–8).

As members of the intermediate filament protein superfamily, keratins are only expressed in epithelial tissue and skin appendages like hair and nails, which are major structural proteins in shear stress resistance and the mechanical support of tissues (9). Keratins currently have 54 members, which are encoded by their own unique genes and are classified into acidic keratins (type I keratins) and basic or neutral keratins (type II keratins) depending on the isoelectric point. There are 28 type I keratins (K9–K10, K12–K28, and K31–K40) and 26 type II keratins (K1–K8, K71–K86) (10). Different types of keratin have similar structures, including a central α-helical rod-like structural domain consisting of approximately 310 amino acid residues, an N-terminal (head) non-helical domain, and a C-terminal (tail) non-helical domain, and are involved in cytoskeleton constitution by binding to each other in heterodimer units that form 10 nm diameter filamentous structures (11). Through connections with desmosomes (cell–cell adhesion) and hemi-desmosomes (cell–matrix adhesion), keratins play important roles in cell polarity maintenance, basement membrane attachment, microstructural organization, material transport, and the regulation of cell migration as well as apoptosis (12–15). Keratin is widely expressed in both normal epithelial tissue and various malignant epithelial tumors. Current research on keratins as tumor diagnostic markers, their mechanisms in tumorigenesis, and as prognostic markers is increasingly prevalent (16). Due to the keratin expression patterns of epithelial malignancies maintaining high similarity with their respective cells of origin, keratins are widely used as reliable tumor diagnostic markers by immunohistochemical assays (17). It has been demonstrated that keratins are also involved in tumorigenesis and progression through various mechanisms, such as mediating multiple signaling pathways and post-translational modifications (16). In addition, there is great clinical promise that keratins can be used as prognostic indicators for various tumors, such as a K5/6-positive phenotype indicating a higher tumor grade and lower survival rate in triple-negative breast cancer (18).

K18 is a member of the type I keratins. Since the 1980s, when Moll et al. proposed the human cytokeratin catalogue, K18 has been focused on and studied continuously as one of the first keratins to be discovered (19). Instead of being located on the long arm of chromosome 17 (17q21.2), like the encoding genes for the other type I members, the K18 gene is found on the long arm of chromosome 12 (12q13.13), along with the type II members (20). The complete structure of the K18 gene has been sequenced, which has a total length of 3791 bp, and seven exons are responsible for encoding the K18 protein. Compared to other keratin genes, the exon structure of K18 exhibits a conserved pattern, except for one exon located at the 3’ terminal. This exon encodes the tail domain of the K18 protein, which in epidermal keratin is responsible for two exons (21). As a non-hair keratin, K18 is usually co-expressed with K8 to form keratin pairs in a 1:1 ratio and involved in the construction of the intermediate filaments of simple epithelial cells in epithelial tissues, such as the hepatocytes, gastrointestinal epithelial cells, mammary epithelial cells, bronchial and alveolar epithelial cells (22). The K8/K18 keratin pairs are mainly localized in the cytoplasmic and perinuclear regions, and if the K8 gene is knocked out the free K18 protein is degraded and keratin intermediate filaments are not formed (23). Therefore, the co-existence of type I (K18) and type II keratins (K8) is necessary for the synthesis and physiological functions of keratin intermediate filaments. The characteristic tissue distribution of K18 and various gene knockout experiments suggest that K18 may play key roles in cellular regulation compared with supportive and mechanical functions (24). It protects cells from apoptosis via auto-glycosylation, strengthens the PI3K/AKT signaling pathway, maintains the placental barrier function, and, interestingly, is involved in the regulation of the cell cycle, which may be related to the phosphorylation of self-specific sites (Ser33) and the binding of adapter/signaling 14-3-3 proteins (25–27). Genetic and spontaneous mutations in keratins are usually accompanied by a variety of visceral diseases, especially liver diseases, like chronic liver disease, primary biliary cirrhosis, chronic pancreatitis, and inflammatory bowel disease (28–30). What is more, K18 is also expressed in cancers of epithelial tissue origin, and, as with other members of the keratin family, it has great potential in tumor diagnosis, prognosis, and tumorigenesis. Currently, the roles of K18 in normal and non-neoplastic diseases have been extensively studied and elaborated. However, the roles and applications of K18 in tumors have not been well summarized. Therefore, this review comprehensively described the current applications of K18 as a tumor diagnostic as well as prognostic marker and summarized the corresponding mechanisms of action with examples of different tumors.

## 2 Keratin 18 as a diagnostic marker in epithelial tumors

In some types of epithelial cells, the sole presence of K8/K18 is a significant feature. For example, K8/K18 is the only keratin pair expressed in hepatocytes. The same characterization applies to other highly specific thin-walled epithelial cells, like pancreatic acinar and islet cells, in addition to proximal renal tubular epithelial cells. In other monolayer epithelial tissues, such as enterocytes and mesenchymal cells, K18 coexists with other simple keratins, such as K7, K19, and K20 (19). At the ultramicroscopic level, these intermediate filaments, composed of simple epithelial keratins, are scattered, and distributed sparsely, which may be related to the weak mechanical stress of the visceral parenchymal epithelium (24). Even in complex or multilayered epithelia (e.g. squamous epithelia and urothelium), K18 may be present with stratified epithelial keratins (31). Therefore, K18 is consistently expressed in various normal epithelial organs. More importantly, K18 is also expressed in tumors of these tissue origins (32). Based on the different cell types, degree of differentiation, and function determining the specificity of keratin expression itself, the epithelial malignant tumors largely retain the specific keratin expression patterns of their corresponding cells of origin even in the epithelial-mesenchymal transition (EMT), in addition to the availability of highly sensitive monoclonal antibodies against K8/K18, the immunohistochemistry of K18 can help identify and diagnose various tumor types (33–35).

K18 is distributed in most epithelial carcinomas. The K18 antibody strongly stains most adenocarcinomas such as breast cancer, lung adenocarcinoma, gastrointestinal adenocarcinoma, and other types of epithelial tumors like hepatocellular carcinomas and renal cell carcinomas (24). Thus, the fundamental application of K18 is to demonstrate the epithelial properties of tumors, especially simple epithelial carcinomas. In contrast, the staining for basal cell carcinomas and differentiated squamous cell carcinomas is typically negative/weak, although Moll et al. observed that K18 expression appears to be more prominent in poorly differentiated squamous cell carcinomas compared to simple epithelial carcinomas in certain cases (33). It is worth noting that K18 expression is not exclusively limited to epithelial cells and may also be present in mesenchymal tumors, such as rhabdomyosarcomas and smooth muscle sarcomas (36, 37). The latter two are often accompanied by other types of intermediate filament expression, like vimentin and desmin (38). Regarding carcinomas of specific organ systems, for example, head and neck carcinomas are divided into two main groups: non-keratinizing squamous cell carcinomas and salivary gland tumors. K18 is currently employed for differentiating the primary sites of various head and neck squamous cell carcinomas, and in salivary gland tumors it serves as a valuable indicator for distinguishing between tumors originating from a stratified epithelium (e.g., basaloid squamous cell carcinoma) and those arising from a simple epithelium (e.g., acinar cell carcinoma) (39, 40). For the sites of origin of pituitary tumors, the anterior pituitary gland (adenohypophysis) is an endocrine gland that expresses simple keratins, whereas the posterior pituitary gland (neurohypophysis) develops from the neural ectoderm as a part of the brain’s gray matter, and thus does not express any keratins (41–43). In cases of lung cancer, the alveolar cells express simple epithelial keratins (K8/K18-positive), while the bronchial epithelial cells exhibit stratified keratin patterns (K5/6, K14, and K17) (44). Pulmonary adenocarcinoma and squamous cell carcinoma are usually considered to be originated from these two types of cells, and Nhung et al. successfully distinguished the two based on their respective keratin characteristics (45). Another study reported that, compared to squamous cell carcinoma and small cell lung carcinoma, adenocarcinoma and large-cell neuroendocrine carcinoma had significantly elevated levels of positive K18 immunostaining (46). In breast cancer, the combination of reduced CK8/18 expression, a basal-like phenotype and a relevant family history may suggest which tumors are associated with BRCA1 germline mutations, thus offering the possibility of simplified genetic testing (47). In liver cancer, hepatocellular carcinoma only expresses K8/K18, whereas hepatobiliary carcinoma is almost always positive for K7 (48). Moreover, adrenocortical tumors are usually benign and negative keratin immunostaining is an important feature of them. However, when adrenocortical tumors display malignant properties, they may express K18 (49).

Therefore, immunostaining for K18 in cancer cells not only aids in determining their epithelial origin but also serves as a differential diagnostic marker for various types of cancers. For some poorly differentiated tumors, or tumors where the primary focus is temporarily unknown but distant metastasis has already occurred, using keratins as markers of pathological diagnosis are significant in determining tumor types and selecting corresponding therapeutic measures early (16). Although in some cases apparent K18 specificity is sufficient to recognize certain cancer types, it is more common that various cancers express similar patterns (50). Therefore, when applying K18 for interpretation it is necessary to consider the impact of atypical tumor behavior and heterogeneity, combining specific expression patterns of other keratins, a patient’s clinical manifestations, and other tumor markers for a comprehensive analysis to achieve a correct cancer diagnosis.

## 3 The roles of keratin 18 in epithelial tumorigenesis

Considering the various discovered regulatory roles of K18, like apoptosis (51, 52), mitosis (27), cell cycle progression (53), and cell signaling (54), in normal cellular physiology, as well as its frequently altered expression (upregulated or downregulated) in cancer, whether K18 plays any functional roles in epithelial tumorigenesis in addition to being a diagnostic marker for the origin of tumors has been extensively investigated. K18 is mainly expressed in simple epithelia, so here the focus is on tumors that arise from them. The expression level and function of K18 are different in different types of tumors.

### 3.1 Lung adenocarcinoma

Lung cancer is the second most prevalent cancer, causing an average of approximately 1.69 million deaths worldwide each year (55). Among these, lung adenocarcinoma (LUAD) is the most common, comprising about 35–40% of all lung cancer patients (56). At present, there are few studies on the specific roles of K18 in lung adenocarcinoma (**Table 1**).

**Table 1:** K18-related proteins in lung adenocarcinoma.

Protein Name	Protein Type	Primary Roles	Modulation of Expression	Reference
EGR1	Transcription factor	Regulate the expression of various tumor suppressors	The expression of K18 is upregulated	(57)
CDK6	Cell cycle regulatory protein	Involved in the G1-to-S-phase transition	The expression is decreased and cell cycle arrest in G1 to S phase	(63)
Caspase-3 and -7	Apoptotic protease	The effectors of cell programmed death	The activity is increased to promote apoptosis	(64)

Zhang et al. reported that K18 expression may be directly regulated by early growth response 1 (EGR1) (57). EGR1 is a nuclear transcription factor that belong to the early response gene family (58). It has been shown to be a tumor inhibitor whose expression is mediated by MAPKs, including the ERK, JNK, and p38 pathways (59, 60). It directly or indirectly upregulates multiple tumor inhibitors, such as PTEN, P53, and TGFβ1, to inhibit cell growth, proliferation, and metastasis, as well as induce apoptosis (61, 62). In Zhang’s study, they found that the K18 promoter contains EGR1 binding sites (EBSs) and demonstrated that EGR1 expression directly upregulates K18 expression through microarray and gene knockout experiments. In K18-overexpressed lung adenocarcinoma cell lines, the expression of CDK6 is decreased and the activity of cleaved-caspase-3 and -7 is increased (57). CDK6 is a cyclin-dependent kinase involved in the G1-to-S-phase transition and enhanced in many cancers, like hematopoietic malignancies, breast cancer, and melanoma (63). Caspase-3, -6, and -7 are involved in the execution of apoptosis as effectors of programmed death (64). In addition, clinicopathological features showed, compared to EGR1-/K18- cases, that the lymph node metastasis of EGR1+/K18+ is significantly lower. These results suggest that K18 may be regulated by EGR1 to act as a tumor inhibitor and reduce the oncogenicity of non-small-cell lung cancer cells. However, another study reported that K18 knockout reduced lung adenocarcinoma cell migration without changes in E-cadherin and vimentin, and increased sensitivity to paclitaxel (65). Therefore, further studies are needed to clarify the specific regulatory mechanisms of K18 in non-small-cell lung cancer progression.

### 3.2 Breast cancer

Breast cancer is the most diagnosed malignancy in women, and the number of women dying from breast cancer ranks second among cancer-related deaths in women (66). It has been demonstrated that K18 plays multiple roles in the progression of breast cancer (**Table 2**).

**Table 2:** K18-related proteins in breast cancer.

Protein Name	Protein Type	Primary Roles	Modulation of Expression	Reference
DR5	Cell surface receptor	Bind with TRAIL to activate intracellular apoptotic signals and induce cell apoptosis	Receptors are restricted to the cytoplasm by autophagy or direct action	(68–70)
LRP16	Coactivator	Enhance ERα-mediated transcriptional activity	Nuclear action is increased and leads to the overactivation of ERα-mediated transcriptional activity	(77)
EpCAM	Transmembrane glycoprotein	Regulate multiple biological processes of cancer cells, including cell adhesion, proliferation, migration, and the EMT	Expression is increased through the activation of the Wnt/β-catenin signaling pathway, to promote the EMT and tumor stem cell features	(82)
Snail	Transcription factor	Inhibits E-cadherin transcription, upregulates the expression of N-cadherin as well as vimentin to promote the EMT, and regulates the expression of the BRCP	The NF-κB/Snail signaling pathway is activated to promote the EMT and MDR	(89)
BRCP	ATP-dependent efflux transporter	Actively excrete antitumor drugs from cancer cells to limit the effect of chemotherapy	The expression is increased to promote the multiple drug resistance of cancer cells	(89)
TGF-β1/TNF-α	Cytokine	Involved in the regulation of immunity and inflammation	The expression of K18 is downregulated under TGF-β1/TNF-α stimulation	(82)
Krtap5-5	Keratin-associated structural proteins	Involved in the regulation of cytoskeletal function	The expression of K18 is downregulated when Krtap5-5 is knocked out	(96)

K8/K18 upregulation protects breast cancer cells from apoptosis. TNF-related apoptosis inducing ligand (TRAIL) transmits apoptotic signals by binding to death receptors (DRs) 4 and 5 on the surfaces of target cells. Fas-associated death domain protein (FADD) and caspase-8 are recruited to assemble death-inducing signaling complex (DISC) to induce the activation of caspase-3 and -7 and ultimately lead to apoptosis (67). The overexpressed K18 breast cancer cells gain resistance to TRAIL-induced apoptotic signals by downregulating DR5 cell surface receptors (68). Although the knockout of K8 increased the protein levels of DR5, the DR5 mRNA did not change. One study reported that K18 was involved in the intracellular clearance of cargo proteins by promoting autophagosome-lysosomal fusion, and another study demonstrated the presence of DR5 in autophagosomes in breast cancer cells, which suggested that K18 may reduce DR5 expression on the cell surface through post-translational pathways mediating autophagy rather than acting at the transcription level (69, 70). DR5 overlapped with K8/K18 and outward diffusion of DR5 from the nuclear and perinuclear regions was observed after knocking down K8, which indicated that K8/K18 may also interact directly with DR5 to restrict its transport (68).

K18 downregulation is involved in the disinhibition of the ERα signaling pathway in estrogen-receptor-positive (ER+) breast cancer. ER+ breast cancer is the most common breast cancer subtype and estrogen receptor alpha (ERα) is a transcription factor nuclear receptor (NR) that plays important roles in stimulating the proliferation and development of mammary epithelial cells (71). ERα is activated by estrogen binding and recruits co-activators together to form an estrogen-binding receptor complex, which act as a transcription factor to regulate the expression of various target genes, such as pS2, cyclin D1, and c-Myc (72). The tumorigenesis of ER+ breast cancer is often associated with the overactivation of the ERα signaling pathway, primarily due to the overexpression of ERα or coactivators (73, 74). Leukemia-related protein 16 (LRP16) is a target gene of ERα and also acts as a coactivator to enhance ERα-mediated transcriptional activity in a ligand-dependent manner, so a positive feedback loop is formed between LRP16 and ERα signaling (75, 76). LRP16 is located in the nucleus to play normal roles. Meng et al. reported that, in MCF-7 cells that overexpressed K18 via transfection, the expression of LRP16 was increased in the cytoplasm with no change in total mRNA levels, and the mRNA levels of ERα downstream genes were significantly reduced under estrogen induced conditions (77). The results suggest that K18 acts as a functional inhibitor of LRP16 by binding and isolating it in the cytoplasm to prevent its nuclear transport, thereby inhibiting the estrogen-induced transcriptional activity of ERα target genes. Therefore, in ER+ breast cancer, the downregulation of K18 leads to the disinhibition of LRP16 nuclear action and the continuous activation of the ERα signaling pathway, to promote the growth and proliferation of breast cancer cells.

K18 downregulation promotes the EMT and multiple drug resistance (MDR) in breast cancer cells. The epithelial-mesenchymal transformation (EMT) is a key process by which epithelial cancer cells acquire the ability to metastasize and promote stem cell formation in various tumors (78). Cancer stem cells (CSCs) are a kind of tumor cell with the ability of self-renewal and differentiation, which play key roles in the onset, invasion, metastasis and drug resistance of tumors (79, 80). During the EMT, the epithelial characteristics disappear and the mesenchymal characteristics are acquired, including motility, invasion, and cytoskeletal remodeling (81). In Shi et al.’s study, compared with the non-metastatic breast cancer cell line MCF-7, the highly metastatic MDA-MB-231 and drug-resistant MCF-7/MX cell lines showed more fibroblast morphology and lower K18 expression (82). When K18 was knocked out the MCF-7 cell line also exhibited similar mesenchymal characteristics—low levels of E-cadherin (the epithelial cell marker) and high levels of N-cadherin and vimentin (the mesenchymal cell markers). What is more, the increased expression of the epithelial cell adhesion molecule (EpCAM) and the reversal of mesenchymal characteristics after continued EpCAM knockout suggested that K18 regulates the EMT through the EpCAM. The EpCAM (also called CD326) is a type I transmembrane glycoprotein and highly expresses in many types of epithelial cancer cells to induce the EMT and promote migration (83–85). Further studies by Shi et al. found that K18 downregulation increased EpCAM expression by activating the Wnt/β-catenin pathway, thus promoting the EMT and cancer stem cell features. Some other studies also support this conclusion (86, 87). In breast cancer, the overactivation of the Wnt/β-catenin signaling pathway is usually associated with high aggressiveness and a low survival rate (88).

Shi’s other study reported that K18 downregulation also activated the NF-κB/Snail signaling pathway to promote the EMT and MDR in breast cancer cells (89). Different from the Wnt/β-catenin signaling pathway mediating the EMT via the EpCAM, the Snail protein directly inhibits E-cadherin transcription and upregulates the expression of N-cadherin and vimentin (90, 91). The breast cancer resistance protein (BRCP) is a member of the ATP-binding cassette transporter family and closely associated with MDR in breast cancer (92). In the MCF-7 cell line, the downregulation of K18 promoted BRCP expression (89). The EMT-mediated tumor resistance has been reported in many studies, and the BRCP promoter has been proved to contain binding sites for transcription factors related to the EMT, including Snail (93–95). Therefore, it can be summarized that the downregulation of K18 activates the NF-κB/Snail signaling pathway to induce the EMT and enhance the expression of BCRP to enable breast cancer cells to acquire MDR.

The breast cancer cells showed mesenchymal phenotypes and the downregulation of K18 under TNF-α alone or TGF-β1/TNF-α costimulation, and, interestingly, under TGF-β1 stimulation alone there was only K18 downregulation without any changes in EMT markers (82). Via the shRNA sequencing of the breast cancer E0771 cell line, Berens et al. reported a newly discovered keratin-associated protein Krtap5-5. Although the expression of K18 was significantly decreased and the expression of mesenchymal markers was increased in Krtap5-5 knockout E0771 cells, this depletion of Krtap5-5 also mediated the downregulation of α6/β4-integrin, ultimately leading to the reduced ability of extracellular matrix invasion and vascular extravasation (96). DNA methylation is an epigenetic mechanism that is used to silence gene expression in normal cells, and abnormal hypermethylation has been observed in many cancers, especially leading to the silencing of tumor suppressor genes and chromosome compaction (97, 98). Azacytidine (AZA) is a methylase inhibitor used in treating many malignant tumors (99). Butler et al. used AZA to treat 231Br brain metastasis breast cancer cells and found that K18 mRNA levels were significantly increased dose dependently, and there was no difference in DNA sequences between 231Br and 231 situ breast cancer cells (100). Therefore, reduced K18 expression may be related to hypermethylation, and it has been found to occur in intron 1 of K18 subsequently. Currently, the exact reasons for K18 downregulation in breast cancer cells remain unclear and these results may imply a possible upstream regulatory mechanism of K18.

Most breast cancer cells present low K18 levels. In summary, a possible explanation for the roles of K18 in breast cancer is that K18 is increased in the early stage of cancer cells to proliferate and avoid immune apoptosis; as the disease progresses it downregulates to promote invasion and metastasis, drug resistance, and malignancy.

### 3.3 Hepatocellular carcinoma

Hepatocellular carcinoma (HCC) is the most common primary malignant tumor in the liver. It is also the fifth most common malignant tumor worldwide, with the second highest mortality rate among all cancers (101). Hepatocellular carcinoma cells retain the unique K8/K18 keratin expression pattern of hepatocytes, and many studies have demonstrated that K18 interacts with various related proteins to regulate the progression of HCC (**Table 3**).

**Table 3:** K18-related proteins in gastric and colorectal cancer.

Protein Name	Protein Type	Primary Roles	Modulation of Expression	Reference
Plectin	Cytoskeletal cross-linked proteins	K18 expression is downregulated and keratin network is disrupted when plectin is knocked out	Dislocate in FA to disrupt integrin-mediated cell migration	(103, 112)
Histone H3	Nuclear protein	Packages DNA into chromatin and regulates gene expression	H3 dysregulation and nuclear polymorphism in hepatocellular carcinoma cells	(106)
RACK1	Scaffold protein	Mediates the localization of FAK	Dislocate in FA to disrupt integrin-mediated cell migration	(112)
PKCδ	Protein kinase	Binds with RACK1 to activate the integrin signaling molecule FAK	Dysfunction to disrupt integrin-mediated cell migration	(112)
Src	Non-receptor tyrosine protein kinase	Modulates the assembly as well as disassembly of FA and the activation of the Rho-ROCK signaling pathway	Integrin-mediated cell migration is disrupted and actin dynamics is impaired	(112–114)
HKII	Metabolic enzyme	Mediates glucose phosphorylation to G6P in glycolysis	The expression is increased to enhance the glycolytic activity	(117)
Glycogen synthase	Metabolic enzyme	Catalyzes UDP-glucose synthesis into glycogen	Activity is increased by activating the mTOR alternative signaling pathway to enhance glycogen synthesis	(117)
Claudin-1	Tight-junction protein	Induces PI3K/Akt membrane activation and regulates MMP2/9 expression	The expression is increased by activating the PI3K/Akt/NFκB signaling pathway to promote tumor invasion and metastasis	(120)
MMP2/9	Protease	Involved in the degradation of extracellular matrix components	The expression is increased to promote tumor invasion and metastasis	(120)
FasR	Cell surface receptor	Binds with Fas ligand to induce apoptosis by activating caspase-8	The membrane localization is increased to promote apoptosis	(120)
sFLIP	Short variant protein	Competitively binds DISC with caspase-8 to inhibit apoptosis	The expression is decreased to promote apoptosis	(120)
PARP	Polymerase	Involved in DNA damage repair	The cleavage is increased to promote apoptosis	(120)

K18 interacts with many structural proteins in hepatocellular carcinoma. Plectin is a multifunctional cytoplasmic cross-linked protein with binding sites for three main cytoskeleton components: microfilaments, microtubules, and intermediate filaments. It acts as a cross-linking agent to connect cytoskeletal components together to form a complete and stable cytoskeletal network (102). One study reported that, in plectin-knocked hepatocytes, K18 expression was downregulated and keratin networks were disrupted (103). In addition, in Ho et al.’s study they found that histone H3 was immunoprecipitated in hepatocellular carcinoma together with K18, while not present in normal liver cells (104). Histones are nuclear proteins responsible for organizing and packaging DNA to form chromatin, and post-translational modifications of histones often alter chromatin structure and regulate gene expression (105). Another study by Cheng et al. reported that the keratin dysregulation caused by plectin deficiency is associated with H3 dysregulation and nuclear polymorphisms in hepatocellular carcinoma cells (106). Therefore, a convincing explanation is that downregulated plectin affects K18 expression in hepatocellular carcinoma tumorigenesis, causing cytoskeletal disturbance, nuclear instability, and vulnerability.

K18 is involved in the integrin-mediated adhesion and migration of hepatocellular carcinoma cells by regulating protein kinase C. Integrin is a transmembrane receptor that mediates the connection between cells and the extracellular matrix (ECM). On the one hand, integrin binds to extracellular signaling proteins to generate intracellular signaling to induce cellular responses; on the other hand, integrin also receives intracellular signaling stimulation to regulate cell movement (107). Cell migration is actually the periodic binding and dissociation between cells and the ECM, which are involved in the dynamic assembly and disassembly of integrin-mediated focal adhesion (FA) (108). FA is a linking complex consists of various proteins, including integrin, PKC, plectin, RACK1, and c-Src (109). There is evidence that a change in FA is regulated by caveolin-1 (Cav-1), which is a substrate of c-Src (110). Therefore, in the cell migration signaling pathway, PKC, as phosphorylated kinase, binds to the anchor-protein-like receptor for activated C kinase 1 (RACK1) to activate the integrin signaling molecule focal adhesion kinase (FAK), and then recruits various proteins to form an FA complex, including c-Src, to complete cell movement. The strict control of cell adhesion and migration is necessary for the normal physiological movement of cells, and uncontrolled regulation often promotes tumor metastasis (111). Galarneau et al. previously found that the absence of K8/K18 in hepatocellular carcinoma disrupts the status of FA (53). In recent studies, by observing the differential migration response of Hev4 (K8/K18-present HCC cells) and shK8b cells (K8/K18-deficient HCC cells) under PMA (a PKC activator) as well as BIM (a novel PKC inhibitor) treatments and PKC knockout experiments, they found that PKCδ is the mediator of the K8/K18 regulation of FA. Further experiments demonstrated that the absence of K8/K18 disrupted FAK activation via dysfunction in PKC, which in turn affected FAK residency in FA, the disassembly and assembly of FA, and cell migration. The loss of K8/K18 as a cytoskeletal protein in hepatocellular carcinoma led to the malposition of plectin and RACK1, as well as affected the activation of the PKC signaling pathway (112). In addition, Bordeleau et al. reported that the loss of K8/K18 also disrupts the activation of the Rho-ROCK signaling pathway, leading to impaired actin cytoskeletal dynamics and cell stiffness (113). Src has been found to be a major regulator of Rho activation, so a possible hypothesis is that the interaction between K8/K18 as an intermediate filament and Rho-mediated actin dynamics may be modulated via the plectin-RACK1-Src of FA (114).

K18 is involved in the regulation of glucose metabolism in hepatocellular carcinoma. Normal liver cells metabolize glucose to produce ATP through the oxidative phosphorylation of pyruvate under aerobic conditions, while hepatocellular carcinoma cells metabolize glucose and produce little energy mainly through anaerobic respiration regardless of oxygen, which is called the Warburg effect (115). Glucose enters the cell via glucose transporters (GLUTs), and its phosphorylation by hexokinase (HK) to glucose-6-phosphate (G6P) marks the onset of glycolysis (116). Compared with H4ev cells, glucose metabolic activity was significantly increased in shK8b cells. GLUT levels did not differ significantly between the two, while HKII showed higher levels in shK8b cells (117). In addition to glycolysis, insulin-induced glycogen synthesis is another important pathway of glucose metabolism in hepatocytes. Insulin binds to the cell surface homologous receptor to activate the PI3K/Akt/GSK-3β signaling pathway, and the phosphorylation of GSK-3β leads to the disinhibition of glycogen synthase to activate glycogen synthesis (118). Interestingly, the activation of insulin-induced glycogen synthase in shK8b cells was demonstrated to occur via the mTOR/S6K alternative pathway and was independent of the PI3K signaling pathway. What is more, the proliferative activity of shK8b cells is increased with insulin stimulation, which is also mediated by mTOR signaling and presents as being glucose-dependent (117). These results suggest that the loss of K8/K18 not only enhances glycolysis during glucose metabolism in hepatocellular carcinoma cells, but also insulin-induced glycogen synthesis and cell proliferation through activating the mTOR signaling pathway.

K18 is also involved in the regulation of the PI3K/Akt signaling pathway to mediate cell motility, invasion, and apoptotic sensitivity in hepatocellular carcinoma. The PI3K/Akt signaling pathway plays important roles in cell proliferation, migration, and anti-apoptosis, and its abnormal transduction is closely related to tumorigenesis (119). Fortier et al. studied the roles of K8/K18 in the PI3K signaling pathway by observing the motility, invasion and drug resistance of human hepatocellular carcinoma HepG2 cell line under different conditions. The phosphorylation levels of PI3K and Akt1/3 were significantly increased in HepG2 shK8/18 cells compared to negative control cells (shNC) ex-pressing scrambled shRNA (120). NF-κB is a transcription factor involved in the ex-pression and maintenance of invasiveness (90). IκBα is an inhibitor of NF-κB, and the PI3K/Akt pathway mediates the phosphorylation of IκBα to induce its degradation. These results suggested that K8/18 knockdown promotes the activation of the PI3K/Akt/NFκB signaling pathway. Although the same signaling protein was observed in breast cancer with Snail upregulation and mesenchymal marker expression, there was no change in HepG2 shK18 cells (89). The aggressiveness of the cells did increase, which was demonstrated by wound healing *in vitro* and transwell assays. Claudin-1, a tight-junction protein, is upregulated in HepG2 shK18 cells and its transcriptional activity is directly regulated by NF-κB. The results of immunostaining and knockout experiments indicated that K8/K18 deficiency enhances the motility and invasion of cancer cells by upregulating the expression of claudin-1 and its localization in the cell membrane and nucleus. The in-creased membrane localization of claudin-1 produced specific regions that induce PI3K/Akt membrane activation, and its increased nuclear localization has been shown to be involved in binding of NF-κB to MMP2 and nine promoters (120). Matrix metalloproteinases are responsible for degrading a variety of protein components in the extra-cellular matrix, such as collagen and elastin. Their abnormal expression can aid tumor cell invasion and metastasis by promoting neovascularization and basement membrane degradation (121). These results revealed that there is a positive feedback loop between the PI3K/Akt/NFκB signaling pathway and claudin-1 to amplify cell motility and invasiveness. Fortier et al. also reported that the knockout of K8/K18 significantly increased the sensitivity of HePG2 cells to cisplatin (120). K8/K18 has been shown to have antiapoptotic effects on stress-induced hepatocytes (52). Although K8/K18 knockout increased the antiapoptotic protein Akt, in the context of cisplatin induction the cleaved caspase-3/8/9 levels and target PARP were also increased to strengthen apoptosis. This apoptotic sensitivity was subsequently demonstrated to be associated with the downregulation of the antiapoptotic protein sFLIP and increased membrane localization of FasR. Claudin1 was found to be participated in the promotion of the localization of FasR on the membrane, which suggested that claudin1 also increases apoptosis sensitivity while promoting cancer cell invasion. Therefore, the loss of the K8/K18 cytoskeleton promotes the movement, invasion, and apoptotic sensitivity of cancer cells by activating the PI3K/Akt signaling pathway.

In addition, K18 may also regulate the progression of hepatocellular carcinoma through some potential pathways, based on its regulatory roles with various related signaling proteins in normal or pathological hepatocytes. As a checkpoint protein, the 14-3-3 protein binds to specific phosphorylated cell cycle regulatory proteins and mediates cell cycle arrest through cytoplasmic sequestration (122). It has been demonstrated that the cellular localization of the 14-3-3 protein is regulated by K18 through binding to the ser33 phosphorylation site (123). K18 has been shown to inhibit TNF-induced apoptosis in simple epithelial cells by specifically binding to the TNFR1-associated death domain protein (TRADD) to block the TRADD binding to activated TNFR1 (124). Apart from these, some studies have also reported the physical interactions of K18 with other regulatory proteins, like p53, NF-κB, and Raf, which dissociate under hyperphosphorylation or stress conditions (125, 126). In conclusion, K18 interacts with various proteins to regulate normal cellular physiological functions, and alterations in these cellular events may contribute to HCC development.

### 3.4 Gastrointestinal cancer

Gastrointestinal cancer refers to malignant tumors occurring in the digestive tract and digestive system, including cancers of the esophagus, stomach, biliary system, pancreas, small intestine, large intestine, rectum, and anus (127). There has been some progress in the roles of K18 in gastric and colorectal cancer (**Table 4**).

According to the Lancet report in 2020, gastric cancer (GC) is the fifth most common cancer and the third leading cause of cancer-related death worldwide (128). Additionally, it has the second highest incidence among gastrointestinal tumors (129). Wang et al. found that K18 was highly expressed in GC cells compared with normal gastric mucosa cells, and its knockout resulted in the inhibition of proliferation and migration, as well as the promotion of apoptosis in GC cells. The total expression levels of MEK, ERK1/2, and c-JUN did not change significantly, while their phosphorylation levels were significantly reduced after K18 knockout, which suggested that the high expression of K18 in GC cells, at least through an excessively activated MAPK signaling pathway, play a promoting role (130). In Chen et al.’s study, they further found that K18 selectively regulated the expression of genes enriched in proliferation and apoptosis processes of GC cells by K18 knockout and RNA sequencing analyses, like NF-κB1, FGF21, and NUPR1. Except transcriptional regulation, K18 has also been reported to regulate the alternative splicing (AS) of multiple genes at the post-transcriptional level, like CDK11A, JNK2, and NCK2, which may be achieved by regulating the expression of splicing factors (SFs) (131). Alternative splicing is an important mechanism for the regulation of gene expression in eukaryotes, which allows a single gene to alternatively splice pre-mRNA to generate multiple mRNA variants, which encode proteins with different functions (132). Incorrect splicing is associated with the development of many malignant tumors, including GC (133). In summary, the altered expression of K18 in GC cells may lead to altered alternative splicing events of the genes involved in proliferation and apoptosis, thereby affecting the structures and functions of various regulatory proteins to promote cancer development.

Colorectal cancer (CRC) was rare in the past; however, due to the influence of population aging, poor diets, and various adverse risk factors, it has been ranked fourth among cancer-related causes of death (134). Like gastric cancer, K18 expression is significantly increased in CRC, and the downregulation of K18 inhibits the proliferation and migration of cancer cells (135). Interestingly, one study reported that the overexpression of K18 enhanced the autophagy capacity and sensitivity to oxaliplatin in colorectal cancer HCT116 cells, and it was reversed after the inhibition of the K18 phosphorylation sites (Ser 33/52A), which suggested that the phosphorylation of K18 is involved in apoptosis regulation in cancer cells (69). The overexpression of K18 in colon cancer cell lines is known to be due to the deregulation of K18 promoter activity, and the mechanism is independent of the binding of factors to specific sequences (136). Prochasson et al. reported that it is associated with the acetylation drive of CBP/p300 and acts on non-histone substrates. Here, they proposed that CBP/p300 may act as an intrinsic component of the pre-initiation complex, without being recruited by specific transcription factors (137). Evidence from other studies showed that the BRCA1/CtIP complex recruits CtBP1 and related deacetylase to the pre-initiation complex to inhibit K18 promoter activity in colon epithelial cells, and this inhibition is altered in colon cancer cells (138). These results revealed that the overactivation of the K18 promoter may be closely related to the acetylation–deacetylation mechanism of the pre-initiation complex and requires further study.

### 3.5 Squamous cell carcinoma

Although K8/K18 pairs were previously thought to be expressed only in simple epithelial cells and its derived epithelial tumors, an increasing number of studies have found that abnormal expression of K8/K18 is also present in various squamous cell carcinomas and is associated with tumor invasion, metastasis, and poor prognosis (24, 139, 140). K8/K18 is induced in cutaneous squamous cell carcinoma, and ectopic co-expression of keratin pairs promotes the invasion of transformed keratinocytes (141). The co-expression of vimentin and K8/K18 was observed in human melanoma tumor cells, and the expression levels of K8/K18 in different cell lines were positively correlated with their aggressiveness (142). Xanthohumol is a newly discovered inhibitor of esophageal squamous cell carcinoma, and K18 has been shown to be one of the targets by reducing K18 expression (143). In terms of the mechanism of K18 mediating the tumorigenicity and progression of squamous cell carcinoma, Alam et al. downregulated the expression of K8/K18 in oral squamous cell carcinoma derived cell lines and found that K18 may be involved in regulating the expression of cell motion-related protein fascin and α6β4 integrin, as well as the phosphorylation activation of downstream signaling molecules such as FAK, Shc, and ERK1/2 to promote tumor invasion and metastasis (144). In addition, the occurrence of these malignant events may also be associated with the loss of phosphorylation of K8 (145).

## 4 Serum level of keratin 18 as a prognostic marker in epithelial tumors

During apoptosis, caspase-3, -7, and -9 are activated as effectors of programmed cell death and cut K18 to form a new epitope, known as M30. During cell necrosis, the integrity of the cell membrane is destroyed and cell contents are released outside the cell without involving the specific cleavage of caspases, resulting in the production of the non-cleaved full-length protein M65. Two sandwiched ELISAs are used to detect different circulating forms of K18 in plasma or serum. The M30 ELISA can only detect cleaved K18 fragments and is therefore used to reflect apoptosis, while the M65 ELISA can detect both full-length K18 proteins and cleaved fragments, thus providing information on the total amount of cell death, including apoptosis and necrosis (146). In laboratory tests, the two methods are often used in combination to distinguish between types of cell death and to assess the relative amounts of apoptosis and necrosis (147). Patients with different types of epithelial-derived cancers usually show high levels of M30 and M65, which are associated with the high proliferative activity of malignant tumors (146). M30 and M65 levels reflect the proliferative ability of cancer cells to a certain extent, and high proliferative activity is often associated with a highly malignant degree of tumors; therefore, measuring serum M30 and M65 levels in patients has the potential to become a simple and effective non-invasive method for predicting or monitoring tumor progression, prognosis, and chemotherapy response.

Many studies have reported the associations between high serum K18 levels and poor prognoses in various tumors, including simple epithelial cell carcinoma and squamous cell carcinoma. In patients with oral squamous cell carcinoma, highly immunoreactive K18 often represents late clinical stage, poor differentiated morphology, and distant metastasis (148). For head and neck squamous cell carcinoma, using microfluidic techniques to detect serum M30 levels of patients can effectively assess cell death induced by low doses of radiation, which may further predict radiotherapy efficacy (149). Compared with normal subjects the serum K18 levels in esophageal squamous cell carcinoma patients were higher and positively correlated with tumor volume, metastasis, and stage (150). In a study by Yaman et al., advanced gastric cancer patients had the significantly increased serum M30 and M65 levels compared to healthy people, and these values were further increased when distant metastasis occurred. Significantly reduced median survival was observed in the patient population with higher M30 levels compared to those with low M30 levels, while serum M65 levels had no effect on survival (147). However, Bilici et al. showed that elevated serum levels of both M30 and M65 were associated with shortened progression-free survival in gastric cancer patients (151). Similar differences were observed in non-small-cell lung cancer. In Ulukaya et al.’s study, patients with high baseline M30 levels had significantly lower median survival compared to patients with low baseline M30 levels (152). In another study, serum total K18 levels showed meaningful results as a prognostic marker, while no significant prognostic value was observed for cleaved K18 (153). In breast cancer, high levels of serum K18 were positively associated with poor prognoses, while tissue K18 expression levels show the opposite conclusion. This may be because the serum K18 level reflects the proliferative activity of tumor cells and the tissue K18 expression level represents the differentiated morphology of tissue, which is consistent with the roles found in tumorigenesis (154). In a study of the evaluation of chemotherapy efficacy in patients with advanced gastric cancer, M30 levels gradually decreased with chemotherapy. Compared to patients who responded to chemotherapy, those who failed chemotherapy tended to have higher serum M30 and M65 levels at the baseline. Additionally, through a stepped-regression analysis, the M30 levels at 14 days of treatment could be used as an independent predictor of efficacy; those patients with lower M30 levels were predicted to have higher rates of progression-free survival and overall survival at a follow-up of one year (155). In colorectal cancer, Koelink et al. reported that the serum M30 and M65 levels in patients with colorectal cancer are associated with disease stages and tumor burden, and the ratio of cleaved K18/total K18 decreased with tumor progression, indicating increased tumor necrosis and worse progression-free survival (156). Tumor necrosis is one of the main causes of systemic inflammation, and systemic inflammation is a stage-independent marker of a poor prognosis of colorectal cancer (157). One study revealed that high levels of total K18, M30, and M65 were associated with high degrees of tumor necrosis, distant metastasis, and elevated markers of systemic inflammation (158). Interestingly, during first-line chemotherapy in patients with metastatic colorectal cancer, total K18 levels decreased first and then gradually increased, which may be related to the apoptosis of chemotherapy sensitive cell populations in early stages and the subsequent increase in chemotherapy resistant cell populations. Therefore, serum K18 levels can be used as an alternative marker of treatment response to monitor chemotherapy efficacy and the development of chemical resistance (159).

Although serum K18 levels show a good correlation with the prognoses of various tumors, the real picture may be more complex. For example, serum M30 and M65 levels were ineffective for predicting neoadjuvant chemotherapy responses in patients with breast cancer, and obstructive jaundice in addition to prolonged plasma placement interfered with baseline K18 levels in patients with pancreatic cancer (160, 161). Therefore, it is necessary to use continuous serum K18 levels in clinical surveillance to exclude confounding factors as much as possible and further evaluate associations in large patient populations.

## 5 Conclusion

K18 plays key roles in a variety of tumors derived from a simple epithelium. As a tumor diagnostic marker, the specific expression patterns of K18 are not only used to determine the epithelial origins of tumors and distinguish different tumor types, but also help to identify the origin cells of some metastatic cancers whose primary sites are unclear (8). The expression levels of K18 are often increased or decreased in tumor cells compared to normal cells, and this change has been demonstrated to play a variety of roles in different tumors, such as promoting invasion and metastasis, anti-apoptosis, and mediating multidrug resistance, which is achieved by the interactions with various related proteins in different signaling pathways (**Tables 1**, **2**, **3**, **4**). In general, with tumor progression, the expression levels of K18 are gradually decreased in liver and breast cancer and increased in gastrointestinal cancer. There is also some conflicting evidence, such as the roles of K18 in lung cancer, suggesting that further research is needed to clarify the specific molecular mechanisms of K18 in different tumors (48, 56). K18 is also abnormally expressed in squamous cell carcinoma, and their underlying clinical significance and role need further study. In addition, due to the high proliferation and death rates of malignant tumors, the serum levels of cleaved K18 and total K18, which represent apoptosis and necrosis, show good correlations with the prognoses of various epithelial-derived tumors. In patients with advanced cancer, high levels of serum K18 are often associated with a high tumor load, late stage, and poor chemotherapy response, which are hopeful to become alternative biomarkers for predicting tumor prognosis.

**Table 4:** K18-related proteins in gastric and colorectal cancer.

Protein Name	Protein Type	Primary Roles	Modulation of Expression	Reference
MEK	Mitogen-activated protein kinase kinase (MAPKK)	Phosphorylation-activated MAPKK phosphorylates MAPK to transmit signals	The phosphorylation level is increased	(130)
ERK1/2	Mitogen-activated protein kinase (MAPK)	Phosphorylated activated MAPK enters the nucleus and phosphorylates transcription factors to mediate transcriptional activity	The phosphorylation level is increased	(130)
c-jun	Transcription factor	Regulates the expression of multiple genes, such as cell proliferation, differentiation, apoptosis, and stress response	The phosphorylation level and transcriptional activity are increased	(130)
Splicing factors	Splicing protein	Removes introns and links exons to form mature mRNA molecules	The alternative splicing events are changed and affect the structures and function of proteins	(131)
CBP/p300	Acetyltransferase	Acetylation drives the expression of target genes	The expression of K18 is upregulated	(137)
CtBP1	Transcription factor	Inhibit the expression of target genes	The inhibitory effect is changed and the expression of K18 is increased	(138)
